# Identification and Validation of a Prognostic Immune-Related Gene Signature in Esophageal Squamous Cell Carcinoma

**DOI:** 10.3389/fbioe.2022.850669

**Published:** 2022-04-13

**Authors:** Kai Xiong, Ziyou Tao, Zeyang Zhang, Jianyao Wang, Peng Zhang

**Affiliations:** ^1^ Department of Cardiovascular Thoracic Surgery, Tianjin Medical University General Hospital, Tianjin, China; ^2^ Department of Thoracic Surgery, The First Affiliated Hospital of Zhengzhou University, Zhengzhou, China

**Keywords:** esophageal squamous cell carcinoma, TCGA, prognostic model, WGCNA, gene signature

## Abstract

Esophageal carcinoma (EC) is a common malignant cancer worldwide. Esophageal squamous cell carcinoma (ESCC), the main type of EC, is difficult to treat because of the widespread morbidity, high fatality rates, and low quality of life caused by postoperative complications and no specific molecular target. In this study, we screened genes to establish a prognostic model for ESCC. The transcriptome expression profiles of 81 ESCC tissues and 340 normal esophageal mucosal epithelium tissues were obtained from The Cancer Genome Atlas (TCGA) and Genotype-Tissue Expression (GTEx) cohorts. The transcriptome expression datasets of 19 esophageal squamous carcinoma cell lines were downloaded from Cancer Cell Line Encyclopedia (CCLE). The R software Limma package was used to identify 6,231 differentially expressed genes and 647 differentially expressed immune-related genes between normal and ESCC tissues. Gene functional analysis was performed using Gene Ontology (GO) and Kyoto Encyclopedia of Genes and Genomes (KEGG). Weighted gene co-expression network analysis (WGCNA) was used to screen out 18 immune-related prognostic genes. We then established the prognostic and risk signature using these genes, and the patients were divided into low-risk and high-risk groups. Compared with high-risk group patients, the low-risk group patients had longer overall survival. M1 macrophages and resting dendritic cells were differentially distributed between the low-risk and high-risk groups and were related to patient survival. We also examined the functional immune cell and immune molecule levels in low-risk and high-risk group patients, with significant differences in the tumor microenvironment between the two groups. To further verify the accuracy of the prognostic risk model, we performed area under the ROC curve (AUC) analysis. The AUC value was 0.931 for the prognostic risk, which was better than the microsatellite instability (MSI) and Tumor Immune Dysfunction and Exclusion (TIDE) scores. In conclusion, we found 18 immune-related prognostic genes related to the occurrence of ESCC and established a prognostic model for predicting disease severity.

## Introduction

Esophageal carcinoma (EC) is a common malignant cancer worldwide. In 2018, about half of all new or fatal global cases of EC originated in China. Esophageal squamous cell carcinoma (ESCC), the main type of EC, is more difficult to treat because it is frequently diagnosed at a late stage (Sung et al., 2021; Wanqing Chen et al., 2021). At present, radiotherapy and chemotherapy are still the main treatments for unresectable advanced EC. Neoadjuvant and molecular targeted therapies are also commonly combined with traditional treatments to extend patient survival (Kakeji et al., 2021). In addition, with the great promise of immune checkpoint inhibitors, such as PD-1, PD-L1, CTLA-4, researchers have started using immunotherapy for advanced EC (Sadanand, 2021). In the KEYNOTE-181 clinical trial study, pembrolizumab had a higher total effective rate and better safety profile compared with chemotherapy. The above results indicated that pembrolizumab should be considered as the new standard of second-line treatment for patients with Combined Positive Score (CPS) ≥10 (Kojima et al., 2020). However, the response rate to EC immunotherapy treatments must urgently be improved to reduce drug resistance and increase patient survival time. The immunotherapy for EC patients is obstructed and long.

For EC patients, especially those with advanced disease, it is of practical significance to better distinguish patients who will potentially respond to immunotherapy. The immune-related gene prognostic index is a recently emerging algorithm that calculates the score of each patient based on the expression levels of related genes. It can be used to predict the patient’s immunotherapy response and prognostic risk index (Yue Chen et al., 2021). In this study, we obtained tumor-related differentially expressed genes by comparing gene expression levels in ESCC tissues and normal esophageal mucosa tissues. We obtained immune-related differential genes by comparing them with immune genes databases. The hub immune-related genes, obtained by weighted gene co-expression network analysis (WGCNA), were used to establish the prognostic and risk signature by immune-related gene prognostic index. Finally, we verified the changes in the immune status of the low-risk and high-risk patient groups and the accuracy of predicting the prognosis and immunotherapy response of ESCC patients.

## Materials and Methods

### Data From the Cancer Genome Atlas, Genotype-Tissue Expression Cohorts and Cancer Cell Line Encyclopedia

The transcriptome expression profiles of 81 ESCC tissues were downloaded from TCGA and the corresponding clinical information (https://portal.gdc.cancer.gov/). The 340 normal esophageal mucosal epithelium samples were obtained from GTEx cohorts (https://www.gtexportal.org/home/index.html). The transcriptome expression datasets of 19 esophageal squamous carcinoma cell lines (TE1, TE4, TE5, TE6, TE8, TE9, TE10, TE11, TE14, TE15, KYSE30, KYSE70, KYSE140, KYSE150, KYSE180, KYSE270, KYSE410, KYSE510, and KYSE520) were downloaded from CCLE (http://www.broadinstitute.org/ccle/home). Using R software to integrate TCGA and GTEx data, 274 normal esophageal mucosa samples and 81 ESCC samples were selected for further analysis. The immune-related gene list was downloaded from the ImmPort dataset (https://immport.org/home).

### Differentially Expressed Genes in TCGA and GTEx Cohorts

The Limma package in R was used to screen the differentially expressed genes in ESCC and normal esophageal mucosa samples. A fold change >2 and *p*-value < 0.05 were the screening conditions (Law et al., 2014).

### Enrichment Analysis

The ClusterProfiler, org. Hs.eg.db, enrichplot, ggplot2, and GOplot packages in R were used to perform functional enrichment analyses of the differentially expressed genes. The Gene Ontology (GO) and Kyoto Encyclopedia of Genes and Genomes (KEGG) analysis results are shown in circular maps (Bindea et al., 2009). The Gene Set Enrichment Analysis (GSEA) was performed to analyze the distribution trend in gene table sorted by phenotypic correlation.

### Screening of Immune-Related Prognostic Genes

The WGCNA package in R was used for the cluster analysis of immune-related differentially expressed genes and screening of prognostic differentially expressed genes. Kaplan-Meier (KM) survival curve analysis was used to further verify the accuracy of the impact of these genes on prognosis (Langfelder and Horvath, 2008).

### CIBERSORT

CIBERSORT is a useful tool for evaluating the abundance of various immune cell types in the tumor microenvironment. A *p*-value < 0.05 was a selective condition, and 73 ESCC samples were obtained for further analysis (Newman et al., 2015).

### Receiver Operating Characteristic Analysis and COX Regression Analysis

The area under the ROC curve (AUC) was used to calculate the predictive ability of the ESCC prognosis model and the COX regression analysis was used to calculate the risk coefficients of ESCC patients. The effect was compared with conventional indexes (Kamarudin et al., 2017).

### Cell Culture

Human ESCC cell lines (TE13, EC109) and normal human esophageal epithelial cells (HEEC) were provided by cardiovascular thoracic surgery department of Tianjin Medical University General Hospital. Cells were cultured with RPMI-1640 containing 10% Fetal Bovine Serum (FBS). All cells were maintained in a humidified chamber containing 5% CO_2_ at 37°C.

### RNA Isolation and Quantitative Real-Time Polymerase Chain Reaction

Total RNA of cultured cells was isolated with RNAiso Plus (TaKaRa) and reverse-transcribed to cDNA with PrimeScript Strand cDNA Synthesis Kit (TaKaRa). Quantitative real-time polymerase chain reaction (qRT-PCR) was performed with TB Green Premix Ex Taq II (TaKaRa). The primer sequences are shown in Supplementary Table S1. Relative gene expression was determined by the comparative 2^−ΔΔCT^ method.

## Results

### Differentially Expressed Genes and Immune-Related Genes in Normal and ESCC Tissues

**FIGURE 1:**
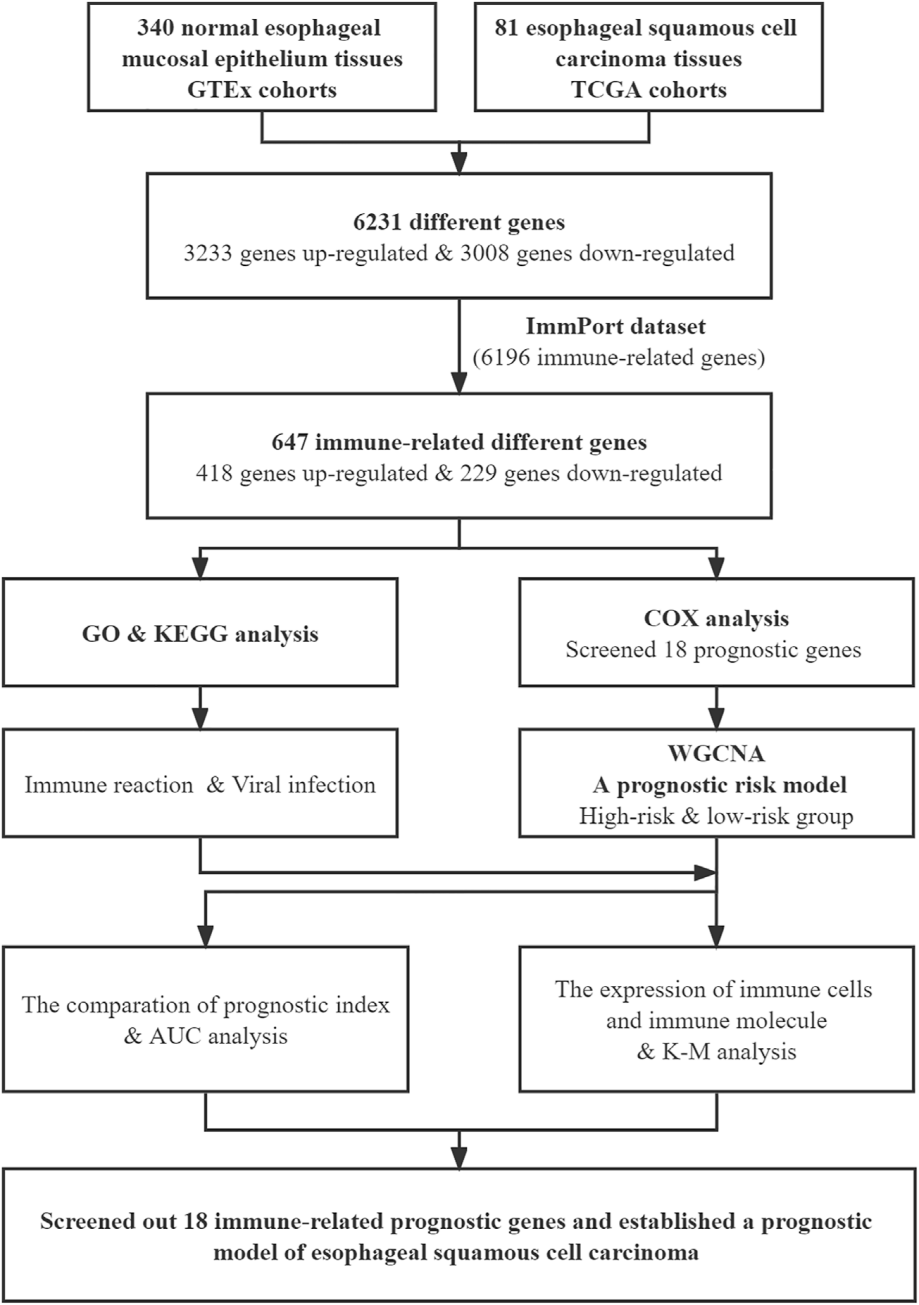
Flow chart of this study.

The flow chart of the study is shown in Figure 1. We downloaded the transcriptome expression profiles of 95 ESCC tissues and 340 normal esophageal mucosal epithelium samples from TCGA and GTEx cohorts, then performed batch integration analysis on the data. Finally, 274 normal esophageal mucosa samples and 81 ESCC samples with 34,350 genes were selected for further analysis (Supplementary Data S1). Differentially expressed genes were screened by Limma. Overall, 6,231 genes were found to be differentially expressed between ESCC and normal tissues. Of these, 3,008 genes were downregulated and 3,223 genes were upregulated in ESCC tissues relative to normal tissues.

**FIGURE 2:**
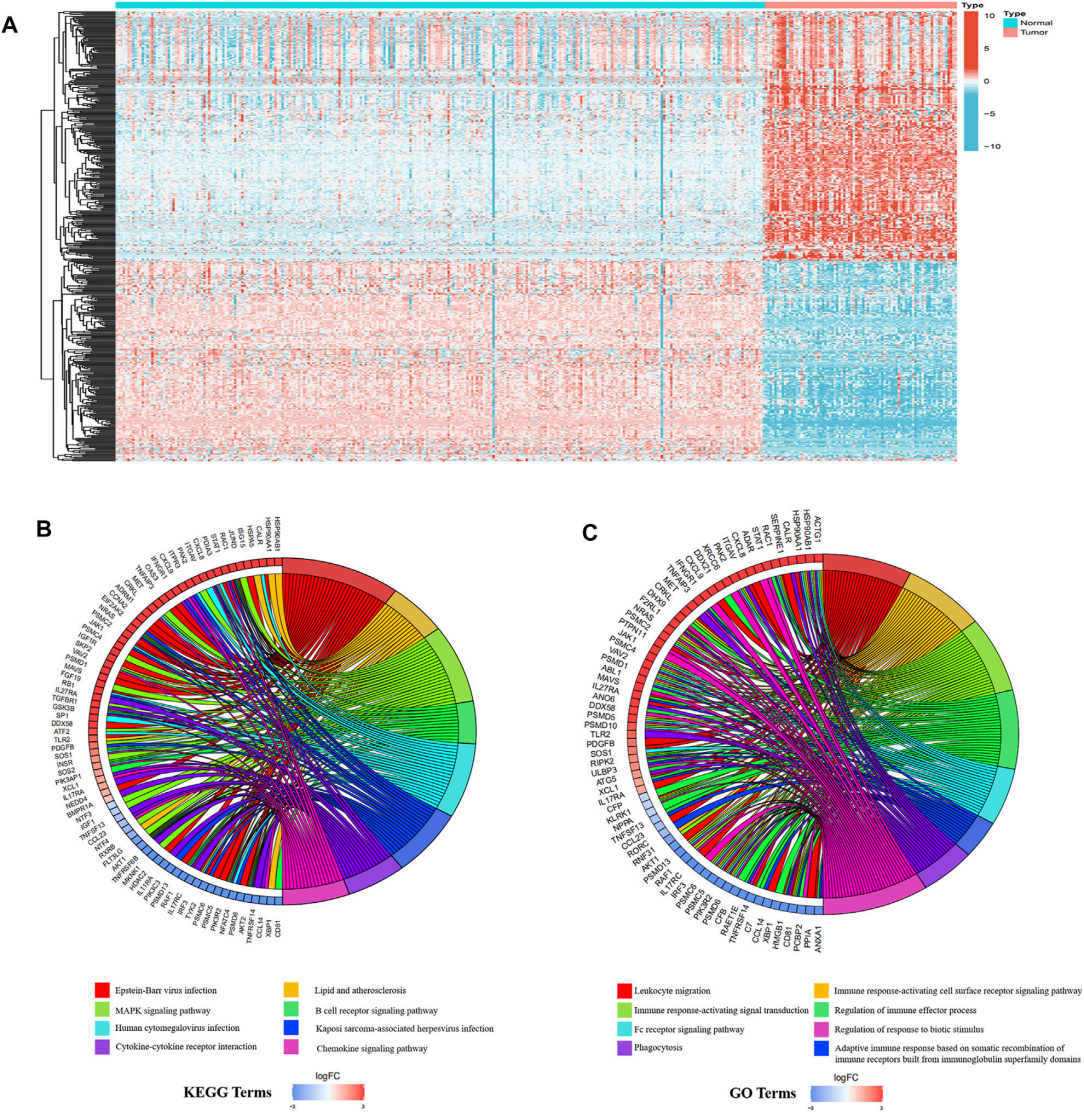
Differentially expressed immune-related genes in ESCC and normal tissues. **(A)** The heatmap of different immune-related genes. **(B)** KEGG analysis results. **(C)** GO analysis results.

Next, we downloaded the list of 6,196 immune-related genes from the ImmPort dataset and screened for any differentially expressed genes among them. There were 647 differentially expressed immune-related genes, with 418 upregulated and 229 downregulated in ESCC tissues relative to normal tissues. The differentially expressed genes are shown in Supplementary Table S2. The heatmaps of differentially expressed immune-related genes and other differentially expressed genes are shown in Figure 2A; Supplementary Figure S1, respectively.

Immune reactions and viral infections may be the main factors that maintain the malignant state of ESCC. GO and KEGG analyses were performed on the differentially expressed immune-related genes, with the results showing that the first eight most significant GO terms enriched were all related to immune response. Additionally, three of the first eight KEGG terms enriched were related to immune response and three were related to viral infection. The enriched KEGG terms, B cell receptor signaling pathway, cytokine-cytokine receptor interaction, and chemokine signaling pathway, were consistent with the enriched GO terms, such as immune response-activating cell surface receptor signaling pathway, immune response-activating signal transduction, and regulation of immune effector process (Figures 2B,C).

### Independent Prognostic Genes Screened by WGCNA in ESCC Patients

**FIGURE 3:**
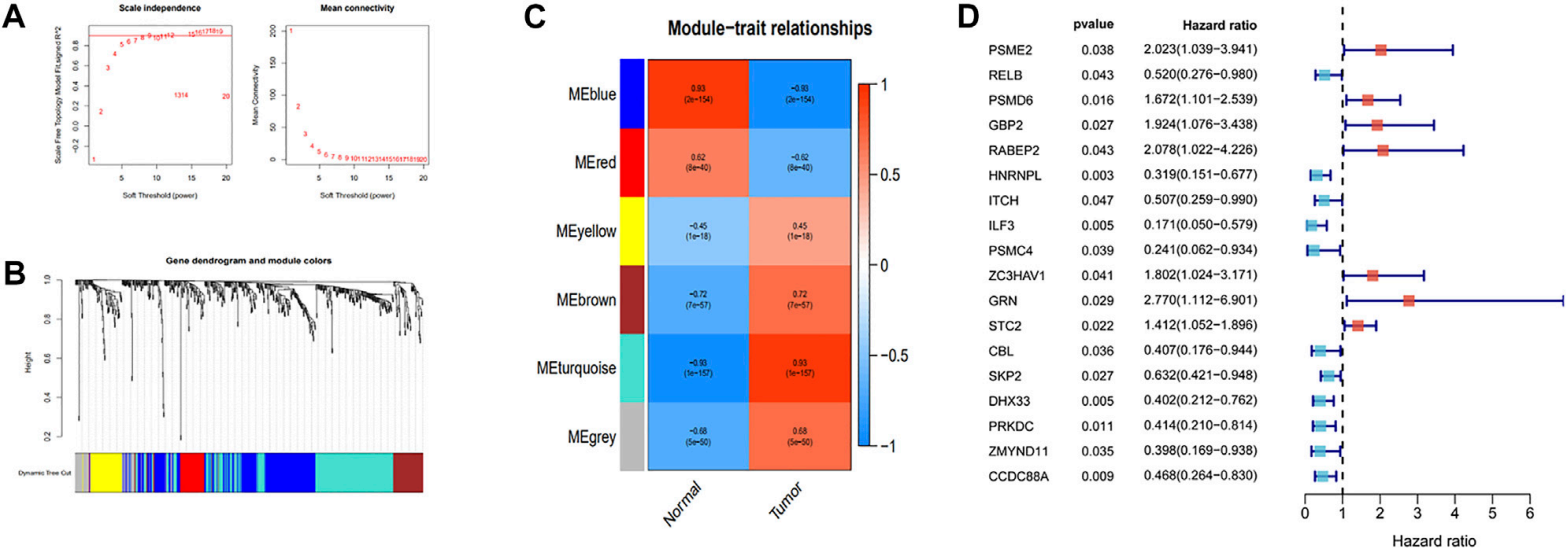
Identification of the independent prognostic genes by WGCNA. **(A)** Analysis of the scale-free index and mean connectivity for various soft-threshold powers. **(B)** Dendrogram of all different genes clustered based on the measurement of dissimilarity. **(C)** The heatmap of the relationship between module eigengenes and tissue types. **(D)** Forest plot of the relationship between gene expression and the risk of survival time.

**FIGURE 4:**
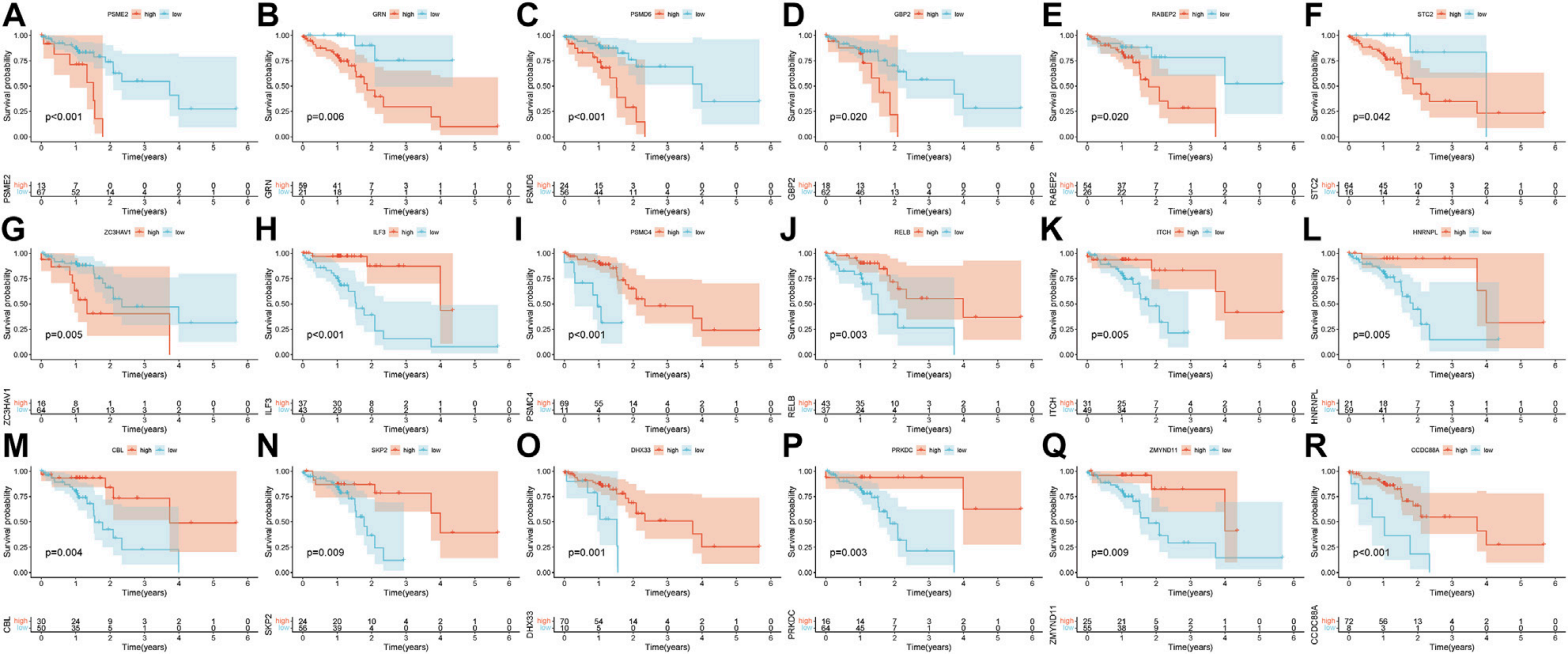
Kaplan-Meier survival curves of the 18 prognostic genes **(A–R)**.

Pearson’s correlation coefficient was used to cluster the immune-related differentially expressed genes in ESCC and normal tissues. The outliers were all removed, and the clustering tree was built (Figures 3A,B). Next, the adjacency matrix and topological overlap matrix were constructed. The six most important modules reflecting the combined function of multiple genes were identified based on the clustering tree and the adjacency and topological overlap matrices (Figure 3C). The blue module with 202 genes and the turquoise module with 222 genes were highly related to the development of ESCC, so these modules were selected for further analysis. Then, we combined gene expression data and patient survival information to calculate the relationship between gene expression levels and days of survival. We identified 18 genes that were related to ESCC patient prognosis, 11 of which were related to good prognosis (*RELB*, *HNRNPL*, *ITCH*, *ILF3*, *PSMC4*, *CBL*, *SKP2*, *DHX33*, *PRKDC*, *ZMYND11*, and *CCDC88A*) and seven that were related to poor prognosis (*PSME2*, *PSMD6*, *GBP2*, *RABEP2*, *ZC3HAV1*, *GRN*, and *STC2*) (Figures 3D, 4A–R).

### A Prognostic Risk Model for ESCC

**FIGURE 5:**
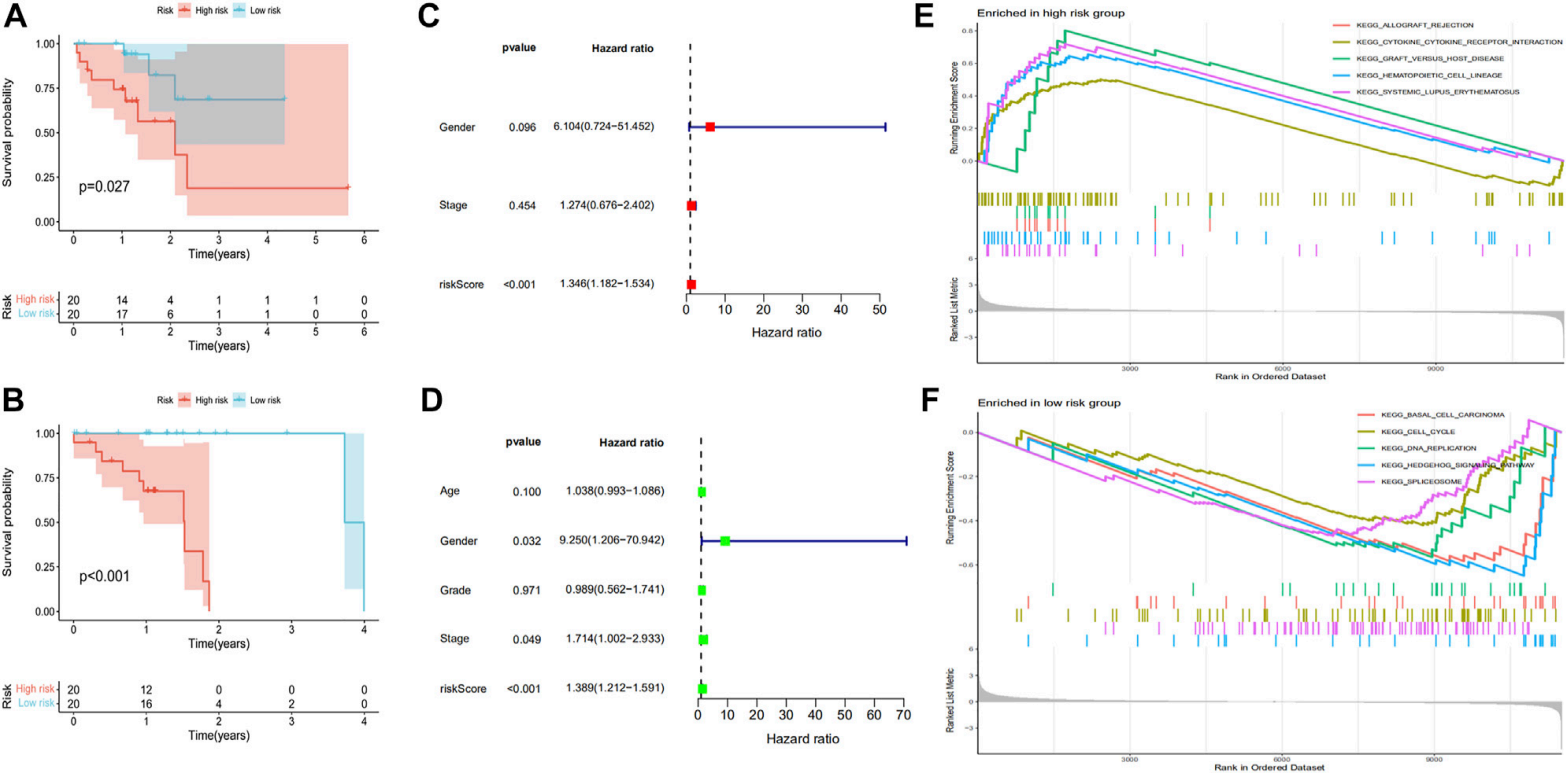
Establishment of a prognostic risk model for ESCC. The Kaplan-Meier survival time was analyzed in the **(A)** training group and **(B)** test group. The independent prognostic analysis was analyzed in the **(C)** training group and **(D)** test group. **(E,F)** show the GSEA enrichment between the high-risk and low-risk groups.

The 81 ESCC selected samples were randomly divided into two groups, training and test. Then, the risk coefficients of 18 prognostic genes were calculated by COX regression analysis. The sum of the product of risk coefficient and expression level for each gene was used to determine the risk degree of ESCC. The R survival package was then used to establish the prognostic risk model. Finally, we found that the prognostic risk model composed of seven of the 18 prognostic genes had high accuracy (*PSMD6*, *RABEP2*, *GRN*, *STC2*, *ITCH*, *ILF3*, and *PSMC4*). The expression levels of *PSMD6*, *RABEP2*, *GRN*, and *STC2* were related to a poor ESCC prognosis, while the expression levels of *ITCH*, *ILF3*, and *PSMC4* were related to a good ESCC prognosis. According to the prognostic risk model, we divided the training and test groups into high-risk and low-risk subgroups. For the training group, patients in the low-risk group had better survival rates, with similar results in the test group (Figures 5A,B). Independent prognostic analyses were then performed on the training and test groups. The results suggested that the risk score was related to ESCC prognosis in both groups (Figures 5C,D). We performed GSEA to verify the accuracy of the established prognostic risk model in predicting ESCC prognosis. There were significant differences in GSEA enrichment between the high-risk and low-risk groups. The GSEA enrichment in the high-risk group was mainly related to immune disease and immune response, while this enrichment in the low-risk group was mainly related to tumor disease and tumorigenesis (Figures 5E,F). The gene mutations between the high-risk and low-risk groups were also different (Supplementary Figure S2).

### The Distribution of Immune Cells and Functional Molecules in ESCC Patients

**FIGURE 6:**
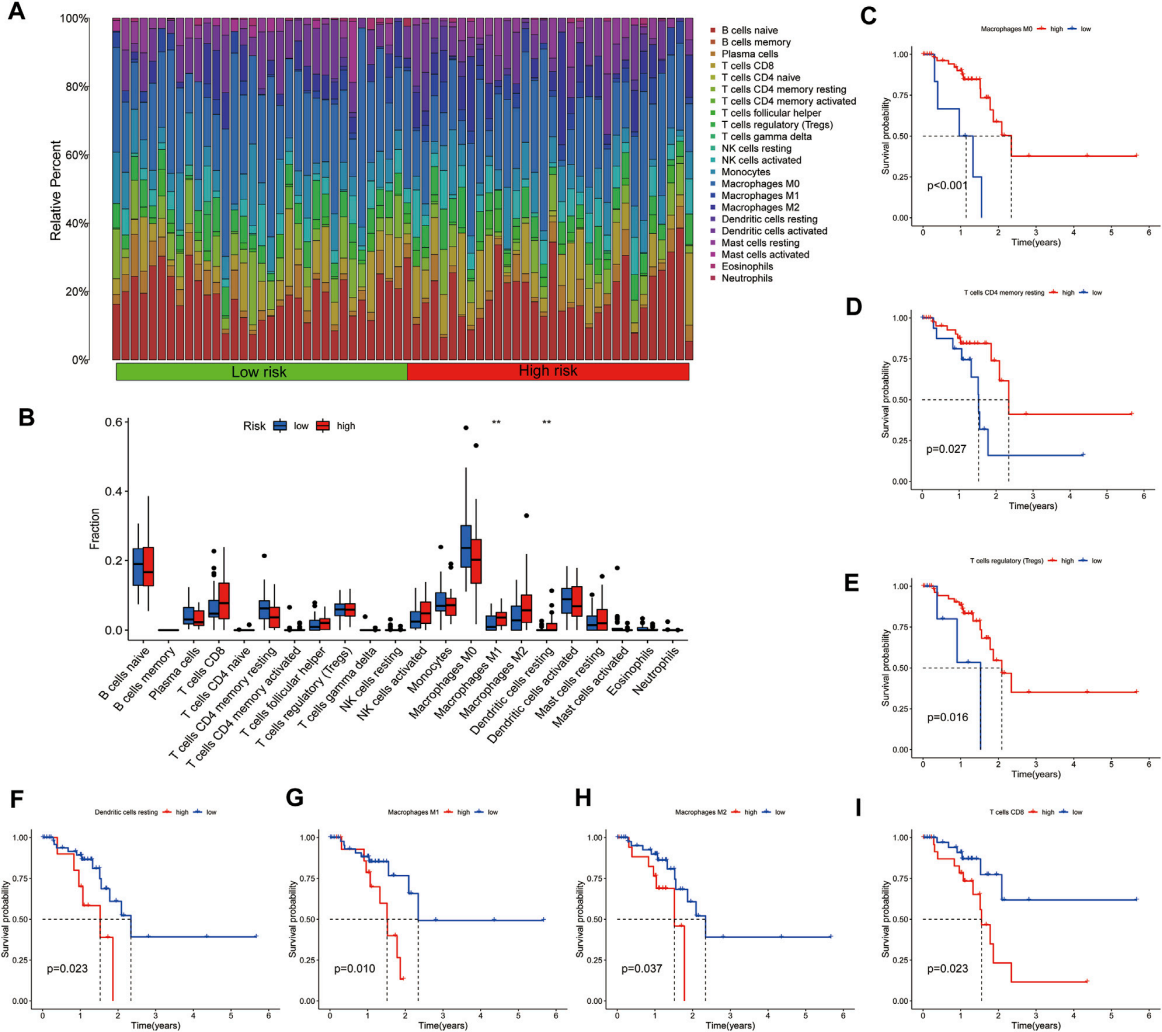
**(A)** Heatmap and **(B)** distribution of immune cells in the low-risk and high-risk groups of ESCC patients. The immune cell types related to good prognosis are shown in **(C–E)**. The immune cell types related to poor prognosis are shown in **(F–I)**.

To examine the immune status of low-risk and high-risk group patients, CIBERSORT was used to evaluate the distribution of 23 immune cell types in the ESCC tumor microenvironment. The proportions of immune cells in the low-risk and high-risk groups are shown in Figure 6A. There was abundant immune cell infiltration in both the high-risk and low-risk groups. We then compared the immune cell composition between these groups, finding increased levels of M1 macrophages and resting dendritic cells in the high-risk groups (Figure 6B). Accordingly, we analyzed the relationship between the immune cell composition and survival time, observing that high levels of resting memory CD4 T cells, M0 macrophages, and regulatory T cells were related to a better survival time (Figures 6C–E). High infiltration of M1 macrophages, M2 macrophages, CD8 T cells, and resting dendritic cells showed opposite effects, suggesting that M1 macrophages and resting dendritic cells were increased in malignant ESCC patients and related to a poor prognosis (Figures 6F–I). Interestingly, high levels of M1 and M2 macrophages were related to poor prognosis, while high amounts of M0 macrophages showed the opposite. These data imply that the different states of macrophages are related to ESCC prognosis.

**FIGURE 7:**
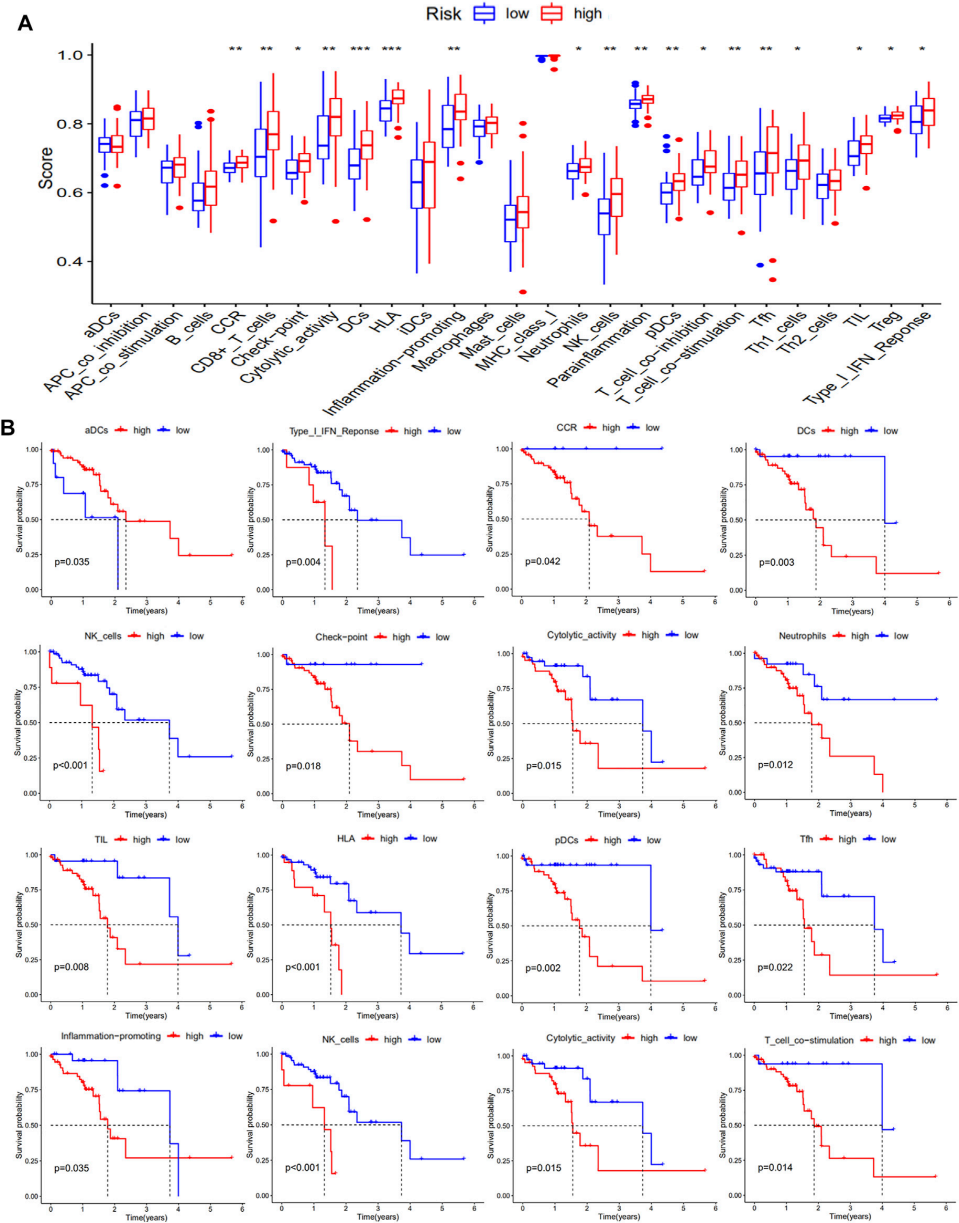
Levels of functional immune cells and functional molecules in ESCC patients. The levels of functional immune cells and functional molecules are shown in **(A)**, and the Kaplan-Meier survival analysis is shown in **(B)**.

The presence of functional immune cells and functional molecules in the high-risk and low-risk groups were also compared. The results showed that a large number of functional immune cells and functional immune molecules were increased in the high-risk group (Figure 7A). Consistent with these data, high levels of functional immune cells and functional immune molecules were related to poor survival. In particular, patients with low levels of Chemokine receptors (CCR), immune checkpoint molecules, plasmacytoid DC (pDCs), dendritic cells (DCs), and T cell co-stimulation had better survival than those with high levels (Figure 7B).

### The Immune-Related Gene Prognostic Index Could Predict the Prognostic Status and Immunotherapy Response of ESCC Patients

**FIGURE 8:**
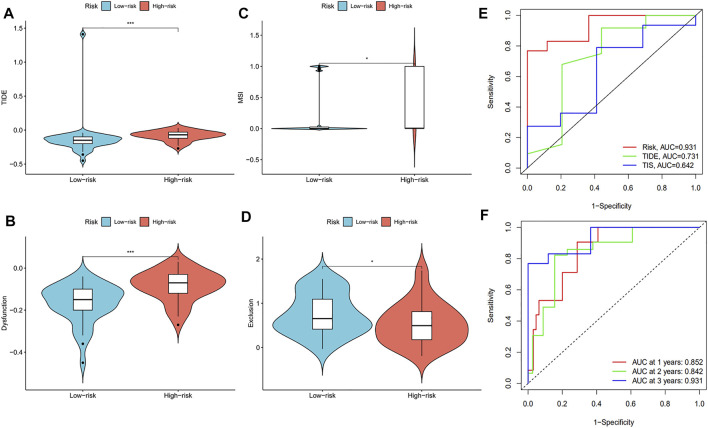
The evaluation of prognostic risk model. The **(A)** TIDE scores, **(B)** dysfunction scores, **(C)** MSI, and **(D)** exclusion scores were analyzed in the low-risk and high-risk groups of ESCC patients. **(E)** ROC analysis of the risk model, TIDE scores, and TIS scores. **(F)** ROC analysis of the 1, 2, and 3-years survival of ESCC patients.

We examined the Tumor Immune Dysfunction and Exclusion (TIDE) scores of the high-risk and low-risk groups to further analyze the difference of immune status between the two groups. The results showed that the TIDE scores were higher in the high-risk group than in the low-risk group (Figure 8A). The dysfunction score of high-risk patients was higher than that of low-risk patients, while the exclusion score showed the opposite trend (Figures 8B,D). These data indicate that the immune escape risk and poor prognosis were higher in the high-risk group. The high degree of microsatellite instability (MSI) in the high-risk group also confirmed this phenomenon (Figure 8C). After comparing the area under the ROC curve (AUC) for the risk score, TIDE score, and Tumor Inflammation Signature (TIS) score, we found the TIDE score to have the best ability of predicting survival time in ESCC patients (Figure 8E). The risk score had a good predictive effect on the 1, 2, and 3-years survival of ESCC patients, and the AUCs were 0.852, 0.842, and 0.931, respectively (Figure 8F). In conclusion, we found 18 immune-related prognostic genes related to the occurrence of ESCC and used several of them to establish a prognostic model for predicting disease severity.

### The Expression Levels of seven Prognostic Genes

**FIGURE 9:**
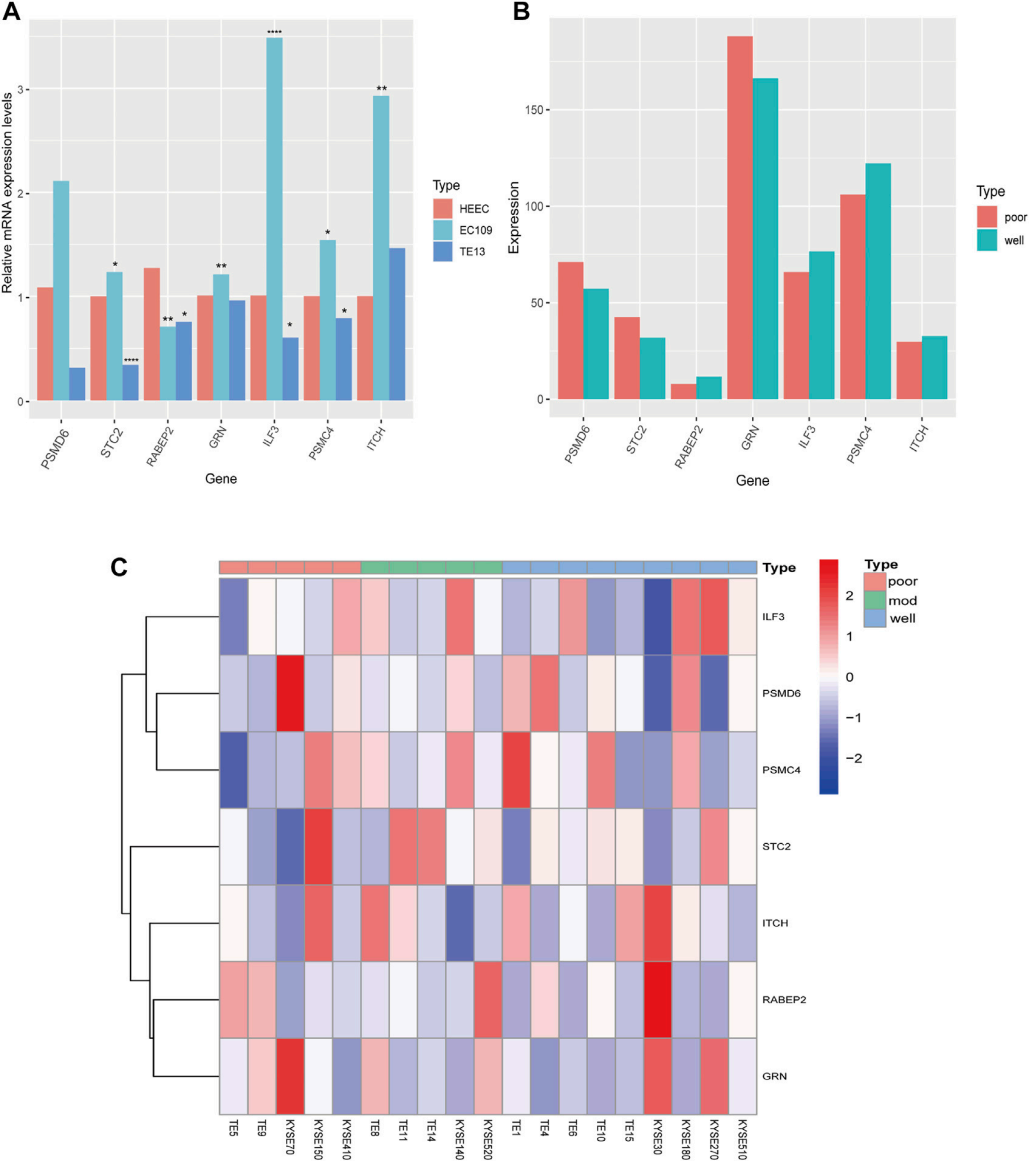
The expression of seven prognostic genes in HEEC and ESCC cell lines. **(A)** The relative mRNA expression levels of seven prognostic genes in HEEC and two ESCC cell lines (EC109, TE13) by qRT-PCR. **(B)** The expression levels of seven prognostic genes in poorly differentiated and well differentiated ESCC cell lines. **(C)** The heatmap of seven prognostic genes expression in 19 ESCC cell lines.

We analyzed the relative mRNA expression levels of seven prognostic genes in the normal human esophageal epithelial cell (HEEC) and two ESCC cell lines (TE13, EC109) by qRT-PCR. As shown in Figure 9A, there were obvious differences in the expression of these genes. Meanwhile, we analyzed the expression of seven prognostic genes in 19 ESCC cell lines in the CCLE database. We found that the expression trends of these seven genes in poorly differentiated (TE5, TE9, KYSE70, KYSE150, KYSE410) and well differentiated (TE1, TE4, TE6, TE10, TE15, KYSE30, KYSE180, KYSE270, and KYSE510) ESCC cell lines were nearly consistent with our previous analysis (Figures 9B,C).

## Discussion

With the emergence of immune checkpoint inhibitor therapies, the treatment options for cancer patients have significantly increased. For ESCC patients, the results of the KEYNOTE-181 clinical trial study highlighted the outstanding achievements of immunotherapy (Kojima et al., 2020). For patients with advanced ESCC, distinguishing the risk degree of individual patients and their response to immunotherapy could better improve the treatment effects. However, there is still no clear molecular targeting index to indicate ESCC severity, nor is there a corresponding prediction model to infer the response of patients to immunotherapy. In this study, we aimed to address this problem by screening prognostic genes and establishing a prognostic immune response model for ESCC. The complex tumor microenvironment is affected by many factors, and the synergistic effect of many genes can affect tumor progression. Through module analysis, WGCNA eliminates the offset of a single factor and obtains important modules related to disease to further analyze important modules and obtain key genes related to prognosis (Langfelder and Horvath, 2008). Using this method, we obtained 18 differentially expressed immune-related genes, 11 of which were associated with a good ESCC prognosis. The other seven genes showed the opposite. Multiple prognostic hub genes still cannot easily prompt the prognosis and risk of ESCC patients. To solve this problem, a prognostic risk model was established using the immune-related gene prognostic index, which could effectively predict the prognostic risk and immune response of ESCC patients (Huang et al., 2021).

The prognostic risk model contained seven genes (*PSMD6*, *RABEP2*, *GRN*, *STC2*, *ITCH*, *ILF3*, and *PSMC4*). PSMD6 encodes a member of the protease subunit 26S family that is part of a multicatalytic proteinase complex with a highly ordered structure composed of two complexes, a 20S core, and a 19S regulator. PSMD6 colocalizes with DNA damage foci and is involved in the ATP-dependent degradation of ubiquitinated proteins, and PSMC4 is involved in the non-ATPase subunits of the 19S regulator lid (Jacquemont and Taniguchi, 2007). The effect of PSMC4 and PSMD6 expression in tumor progression is still unclear. RABEP2 is a recently identified protein required for the formation of collateral vessels during development, which is possibly why it can promote the development of an ESCC tumor (Aghajanian et al., 2021). GRNs are a family of secreted, glycosylated peptides that are cleaved from a single precursor protein with 7.5 repeats of a highly conserved 12-cysteine granulin/epithelin motif. Research has suggested that GRN can promote ESCC progression *via* the autocrine-dependent FAM135B/AKT/mTOR signaling pathway (Dong et al., 2021). STC2 encodes a secreted homodimeric glycoprotein that is expressed in a wide variety of tissues and may have autocrine or paracrine functions. A previous study regarded STC2 as a predictive marker for lymph node metastasis in ESCC (Kita et al., 2011). Moreover, it was reportedly involved in breast, gynecologic, gastric, colorectal, liver, and respiratory cancers (Li et al., 2021). ITCH is a regulator of lymphocyte differentiation and the immune response. Mutations in this gene are involved in the development of multi-system autoimmune diseases (Kleine-Eggebrecht et al., 2019). Studies have reported that circulating ITCH may have an inhibitory effect on ESCC by regulating the Wnt pathway (Yan et al., 2020). ILF3 has been reported to contribute to the development of various cancers (Hu et al., 2013; Cheng et al., 2018; Liu et al., 2019). High expression of ILF3 was shown to be involved in the metabolic alterations in ESCC patients, especially for the intermediate metabolites of the glycolysis pathway (Zang et al., 2021). Through survival analysis, we found that the expression of *PSMD6*, *RABEP2*, *GRN*, and *STC2* were related to a poor ESCC prognosis, while the expression of *ITCH*, *ILF3*, and *PSMC4* were related to a good prognosis. The immune prognosis analysis model established with these genes was reliable.

According to this immune prognostic risk model, we divided ESCC patients into low-risk and high-risk groups. We then analyzed the relationship between immune cells and the degree of malignancy of ESCC patients. We found the level of resting DCs to be elevated in malignant ESCC patients and related to poor prognosis. DCs are considered to be key antigen-presenting cells (APCs) that are mainly used to control the initiation of T cell-dependent immune responses (Saiz et al., 2018). Resting DCs are present in a variety of tissues, with their main role being to bind and internalize antigens. However, their role in antigen presentation and activation of T cells is limited. The transformation of resting DCs into activated DCs is affected by a variety of factors that regulate the expression of costimulatory and adhesion molecules. Through this process, the capacity for antigen uptake is lost and the ability of potent T cell stimulation is acquired (Montoya et al., 2002; Probst et al., 2005; Steptoe et al., 2007). Studies have shown that resting DCs are abnormally distributed in a variety of tumors, including clear cell renal cell carcinoma (Pan et al., 2020), colorectal cancer (Li et al., 2020), lung adenocarcinoma (Zhang et al., 2021), and colon cancer (Cui et al., 2021). This also illustrates the important role of resting DCs in tumorigenesis and development.

As a plastic and pluripotent cell population, macrophages exhibit significant functional differences under the influence of different microenvironments *in vivo* and *in vitro* (Artyomov et al., 2016). According to the activation state and function, macrophages can be divided into M1 and M2 (Yunna et al., 2020). M1 type macrophages secrete pro-inflammatory cytokines and chemokines and participate in the positive immune response. M2 type macrophages have only weak antigen presentation ability and downregulate the immune response by secreting inhibitory cytokines such as IL-10 and TGF-β (Orecchioni et al., 2019; Anderson et al., 2021). Our results show that the various macrophage stages have different effects on the prognosis of ESCC patients, which is consistent with the previously described findings in the field. Therefore, the distribution of immune cell types can affect the disease malignancy of ESCC.

The TIDE score is an advanced method to predict the sensitivity of tumors to immune checkpoint treatment by using gene expression information. The TIDE score calculated by the software consists of two parts: dysfunction score and exclusion score. The calculation principle of the dysfunction score is that genes with immune disorders have a higher weight, and it can be obtained by multiplying by the expression level. The exclusion score is obtained by multiplying the weight of immune rejection genes by the expression level (Wang et al., 2020). The TIDE score has been confirmed to have higher accuracy in evaluating the efficacy of PD1 and CTLA4 monoclonal antibody treatment (Kim et al., 2021). The TIS score was an important index to evaluate the reactivity to PD1 monoclonal antibody treatment by calculating the levels of inhibited T cells in the tumor microenvironment (Danaher et al., 2018).

MSI refers to the phenomenon that new microsatellite alleles appear in the tumor from a change to the microsatellite length caused by the insertion or deletion of a repeat unit (Vilar and Gruber, 2010). MSI has been widely confirmed in colon cancer (Boland and Goel, 2010), and its mechanism mainly relies on the lack of a DNA mismatch repair system. MSI has also been detected in other cancer types, including gastric cancer (Ratti et al., 2018), endometrial cancer (Latham et al., 2019), and ovarian cancer (Evrard and Alexandre, 2021). TIDE, TIS, and MSI are classic indicators of tumor microenvironmental immunity and tumor evaluation, with TIDE and TIS focusing on T cell function and MSI focusing on genetic changes. However, these indicators could not fully reflect the complex microenvironment of the tumors. In our study, compared with the TIDE and TIS scores, the immune prognostic and risk model was a better predictor of survival time. This model is possibly a better prediction method for the prognosis and immune response of ESCC patients.

## Conclusion

Here, we found 18 immune-related prognostic genes related to the occurrence of ESCC and used several of them to establish a prognostic model for predicting disease severity.

## Data Availability

The original contributions presented in the study are included in the article/Supplementary Material, further inquiries can be directed to the corresponding author.
