# Supplementary figures and images for: Identification and Validation of a Prognostic Immune-Related Gene Signature in Esophageal Squamous Cell Carcinoma
Source: Front Bioeng Biotechnol. 2022 Apr 13;10:850669. doi: 10.3389/fbioe.2022.850669 (PMC9043362; doi:10.3389/fbioe.2022.850669)

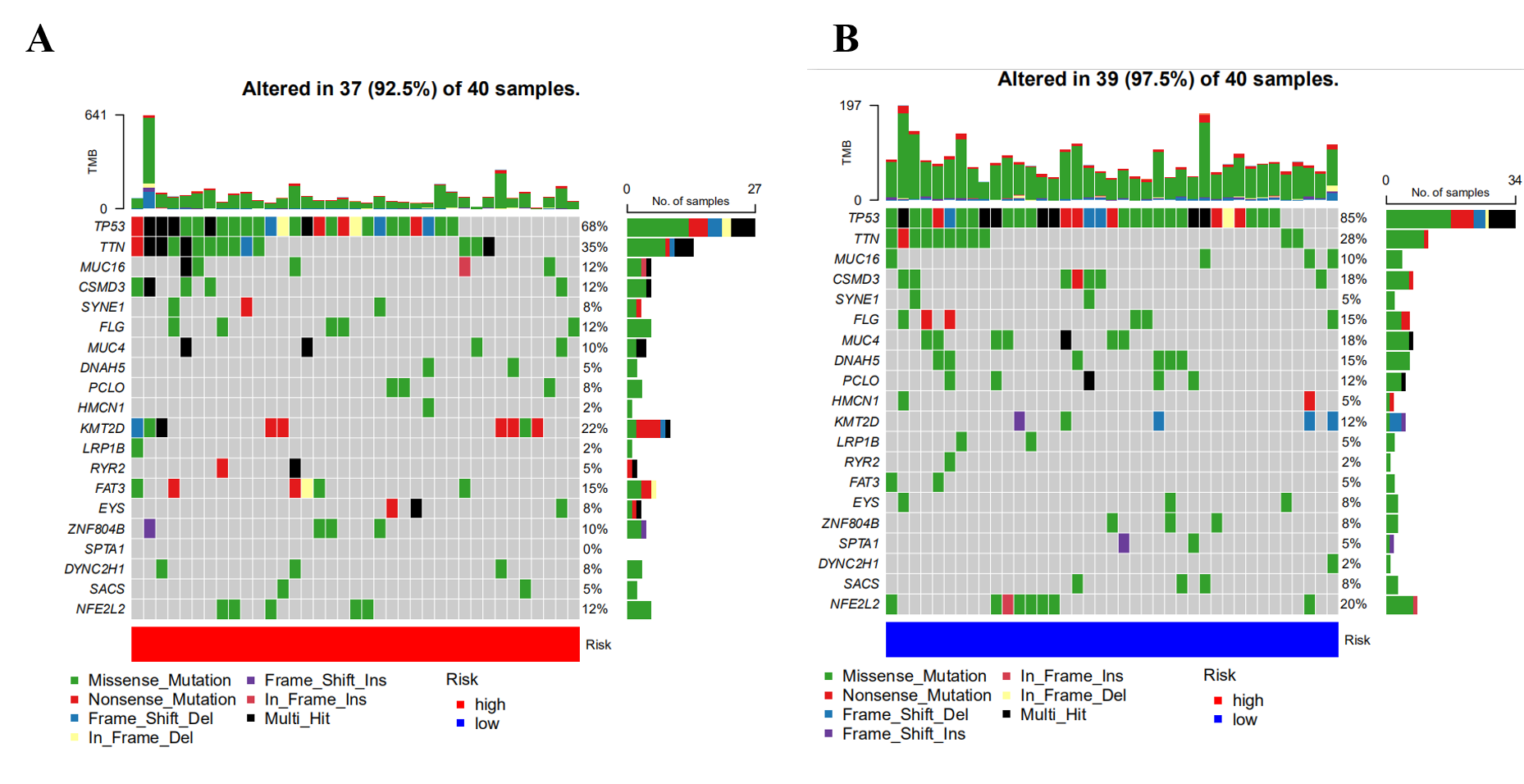

Supplement: Supplementary file 3 [file Image2.TIF]

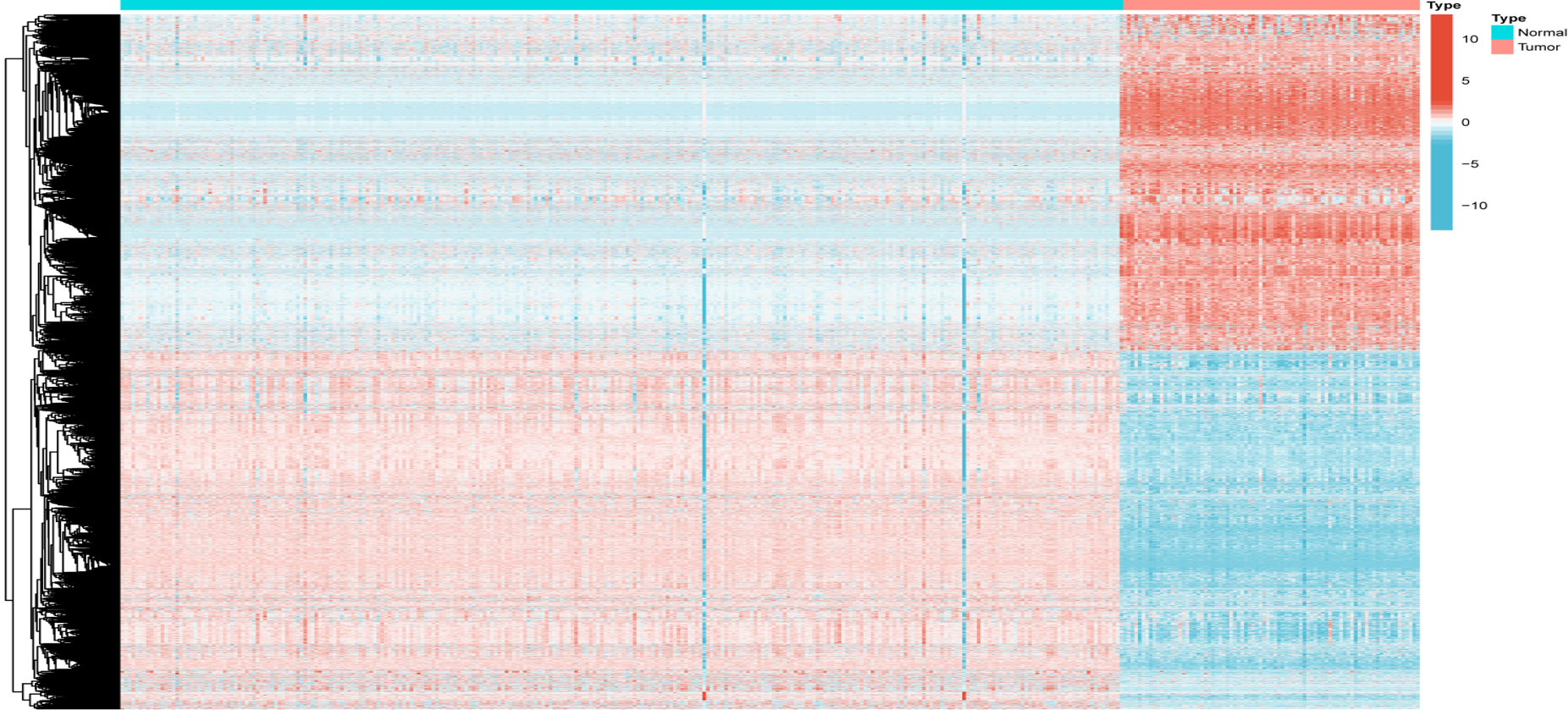

Supplement: Supplementary file 4 [file Image1.TIF]
